# The introduction of fluoroscopic surgery: A report of an initial trial case

**DOI:** 10.1016/j.ijscr.2023.109202

**Published:** 2024-01-10

**Authors:** Junji Takahashi, Masashi Yoshida, Teppei Kamada, Keigo Nakashima, Norihiko Suzuki, Yutaka Suzuki

**Affiliations:** Department of Surgery, International University of Health and Welfare, Tochigi, Japan

**Keywords:** Fluoroscopic surgery, Fluorescent angiography, Indocyanine green, Laparoscopic surgery

## Abstract

**Introduction:**

Switching from white light to fluorescence mode is necessary to confirm the fluorescence during fluorescence-guided surgery. This case report presents the use of a syringe pump to continuously inject indocyanine green (ICG), which enabled the vessels to be visualised and the operation to be performed without switching.

**Presentation of case:**

An Asian male patient in his 40s underwent an interval appendectomy following conservative treatment for appendicitis. Laparoscopic surgery was performed using the VISIONSENSE® system. Diluted ICG (25 mg/15 mL) was intravenously administered at 1 mL/min.

The appendiceal artery was visualised in light green, and the intensity of the visualisation was defined relative to the tissue surrounding the dissected appendiceal artery. The superior rectal artery and the vessels within the mesentery of the small intestine were confirmed to be continuously visualised throughout the surgery.

Therefore, continuous ICG angiography made it possible to operate while keeping the appendiceal artery visible in this case.

**Discussion:**

ICG angiography enabled the operation to be performed with the appendiceal artery continuously visualised. This method was developed for use in cancer surgery; however, since operations of longer duration are speculated to require larger doses of ICG, we opted to introduce this method in an initial trial for appendectomy.

**Conclusion:**

The fluoroscopic surgery using a syringe pump was feasible in this first case report without switching to white light mode.

## Highlights

• We report a fluoroscopic surgery technique where ICG was continuously infused.
• This is the first study to use continuously infused ICG during surgery.
• The appendiceal artery remained visible while using continuous ICG angiography.

## 1 Introduction

The surgical procedure that uses the advantage of fluorescence is known as “fluorescence-guided surgery”. Switching from white light to fluorescence mode is necessary during fluorescence-guided surgery to confirm the fluorescence. In such cases, the surgical procedure is stopped while confirming the fluorescence. However, the development of the bright field full-colour fluoroscope enabled us to perform indocyanine green (ICG) fluorescence-guided laparoscopic cholecystectomy without switching to white light mode, and we reported the first case [1]. Therefore, this type of fluorescence-guided surgery without switching to the white light mode was termed “fluoroscopic surgery”.

The use of ICG fluorescence techniques for intraoperative vessel visualisation has been suggested as a valuable technique in neurosurgery and reconstructive surgery [2,3]. Moreover, its utility has been reported in gastric and colorectal cancer surgeries to identify the subpyloric artery [4] and target vessels [5], respectively. Typically, ICG is administered through a single intravenous bolus injection, making arteries visible within 1 min.

However, an obstacle to performing fluoroscopic surgery for vascular and blood flow recognition was the rapid disappearance of ICG from the blood flow. Approximately 90 % of ICG disappears from the blood flow within 15 min, and the arteries are only clearly visualised in the first 30 s [6].

Therefore, to resolve this challenge, we attempted to administer ICG continuously using a syringe pump. The first trial of this new fluoroscopic surgery using a syringe pump was applied in this case of appendectomy, a procedure usually completed within 1 h.

This report was prepared in accordance with the SCARE 2023 criteria [7].

## 2 Presentation of case

An Asian male patient in his 40s with diabetes mellitus (glycated haemoglobin level of 7.7 %) and chronic renal failure underwent treatment with dialysis. Two months after conservative appendicitis treatment, the patient underwent an interval appendectomy with laparoscopic surgery performed by a resident physician.

**Fig. 1:**
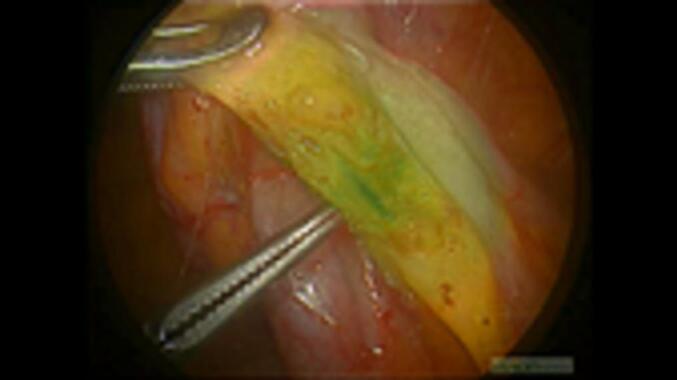
Blood vessels are continuously illuminated in green. (For interpretation of the references to colour in this figure legend, the reader is referred to the web version of this article.)

The VISIONSENSE® laparoscopic system (Medtronic, Dublin, Ireland) was employed in this laparoscopic surgery. However, we did not use the threshold-adjustment function of this system to discard the near-infrared signal or the infrared boost function. Diluted ICG of 25 mg/15 mL was administered intravenously using a syringe pump at 1 mL/min and continued for 7.7 min until the end of the vascular dissection (Fig. 1).

The appendiceal artery was visualised in light green and was clipped and dissected with an energy device for 1.67 min (Fig. 1). The appendiceal vein appeared darker green than the appendiceal artery, and the visualisation intensity was defined relative to the tissue surrounding the dissected appendiceal artery.

**Fig. 2:**
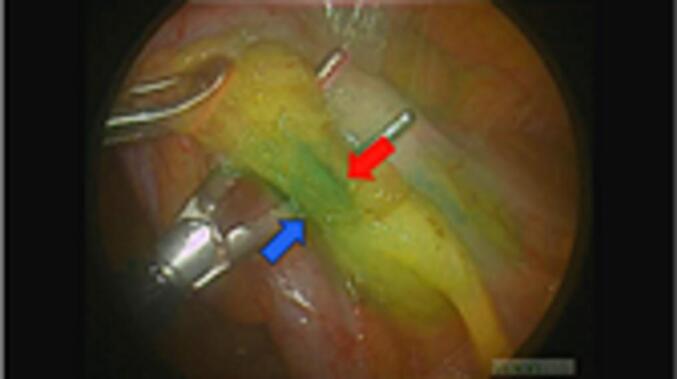
The appendiceal vein (blue arrow) appeared darker green than the appendiceal artery (red arrow). (For interpretation of the references to colour in this figure legend, the reader is referred to the web version of this article.)

**Fig. 3:**
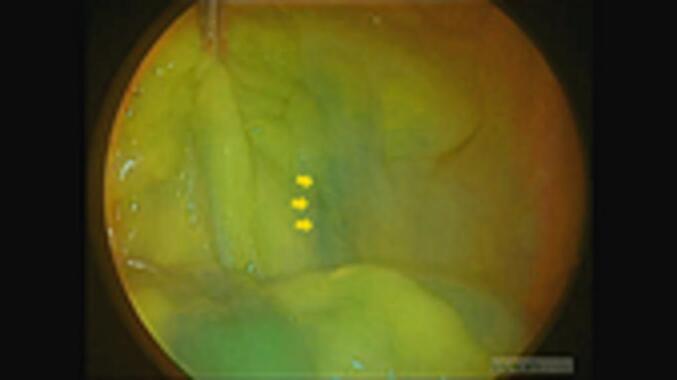
The superior rectal artery (orange arrows) was confirmed to be continuously visualised throughout the surgery.

**Fig. 4:**
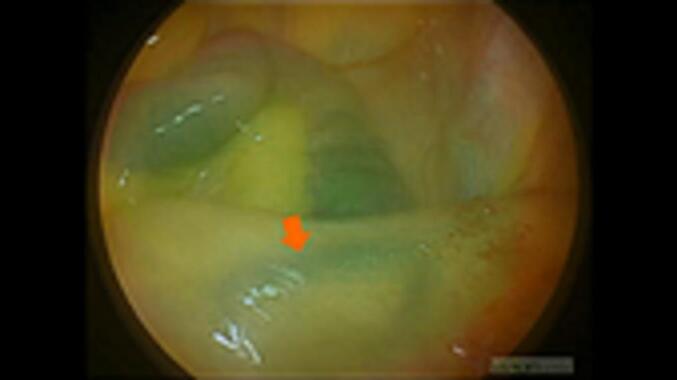
The vessels within the mesentery of the small intestine (red arrow) were verified as fluorescent through to the end of the operation. (For interpretation of the references to colour in this figure legend, the reader is referred to the web version of this article.)

Subsequently, we ligated the appendiceal vein at the level of the appendiceal root and dissected it using an energy device (Fig. 2). The appendix was removed and assessed for intra-abdominal bleeding. Simultaneously, the superior rectal artery and the vessels within the mesentery of the small intestine were confirmed to be continuously visualised under fluorescent angiography (Fig. 3, Fig. 4). The total operative time was 56 min, with 5 g of blood loss; however, no intraoperative complications were observed. Finally, the patient was subsequently discharged from the hospital uneventfully 2 days after surgery.

## 3 Discussion

This is the first report of fluoroscopic surgery of the gastrointestinal tract.

The concept of fluoroscopic surgery was inspired by the development of fluorescent cameras, which were initially launched as monochrome cameras [8] and later advanced to full colour, applied to laparoscopy, and advanced to dichromatic [9]. Currently, fluorescent laparoscopic cameras are equipped with bright-field and full-colour capabilities [10], including the recently developed high-definition technology. Therefore, using this continuously fluorescing device, we could assess what exhibits fluorescence and what does not and reach a level where surgery may be improved by viewing fluorescent images.

In this case, the continuous ICG angiography enabled us to operate while keeping the appendiceal artery visible. Continuous ICG angiography for the location of vessels can be performed even when the intestinal tract is moved, allowing surgery to be performed with a constant awareness of the artery's location, including unexpected anomalies. The tissue around the vessel was successfully dissected while outlining the vessel diameter, potentially reducing the risk of accidental vessel injury.

This initial demonstration of continuous ICG angiography shows that continuous ICG can be used instead of a single intravenous bolus injection. Furthermore, since we could continuously observe the superior mesenteric artery and small mesenteric vessels, it also provides the possibility that this method can be applied to other areas of surgery in the future.

However, since this is only one case, it should be noted that visibility may be insufficient in cases with a thick mesangial fat layer; therefore, a case series should be conducted to examine the efficacy of this method in several types of surgery.

## 4 Conclusion

The fluoroscopic surgery using a syringe pump without switching to white light mode was feasible in this first case report.

## Informed consent

Written informed consent was obtained from the patient for publication and any accompanying images. A copy of the written consent is available for review by the Editor-in-Chief of this journal on request.

## Ethical approval

Ethical review was not required for this case report as it was a study using an existing drug and a single case report.

## Funding

We have no sponsors.

## Author contribution

JT: study design, data collection, data analysis, writing.

MY: critical revision

YS: final approval of the article

Any other authors: study design, data collection

All authors read and approved the final manuscript.

## Guarantor

Junji Takahashi, the corresponding author of this manuscript accept full responsibility for the work and the conduct of the study, access to the data and controlled the decision to publish.

## Research registration number

This paper is case report. The authors don’t need to register this work.

## Conflict of interest statement

There are no conflicts of interest.
